# Healthcare workers’ views and actions on climate change and health in private healthcare facilities in Tanzania: a cross-sectional study

**DOI:** 10.1136/bmjopen-2026-117293

**Published:** 2026-07-28

**Authors:** Sophia Lubna Kara, Ahmed M Jusabani, Elishah Premji, Muzdalifat Abeid

**Affiliations:** 1 Ottawa Hospital Research Institute (OHRI), Ottawa, Ontario, Canada; 2 Aga Khan Hospital Dar es Salaam, Dar es Salaam, Tanzania

**Keywords:** Climate Change, Health Services, Health Workforce, Africa South of the Sahara, PUBLIC HEALTH

## Abstract

**Abstract:**

**Objective:**

To assess the knowledge and attitudes of clinical and non-clinical healthcare workers related to climate change and health in Tanzania and to explore their roles in supporting sustainable healthcare practices.

**Design:**

Multicentre, cross-sectional survey.

**Setting:**

Private healthcare facilities across multiple levels of care in Tanzania.

**Participants:**

Healthcare workers employed at participating private healthcare facilities. A total of 216 respondents completed the survey, including both clinical (65.7%) and non-clinical (34.3%) staff.

**Primary and secondary outcome measures:**

Primary outcomes included healthcare workers’ knowledge and attitudes regarding climate change and health, as well as their awareness of the healthcare sector’s role in environmental degradation. Secondary outcomes included barriers to workplace climate action and existing engagement in environmentally sustainable healthcare practices.

**Results:**

Over 90% of respondents recognised climate change as a significant health threat, and nearly 80% acknowledged that healthcare facilities contribute to environmental impacts. Major barriers to sustainable practices included low awareness among colleagues, budget constraints and a perceived lack of institutional or leadership support. Reported actions included responsible energy use, waste and water management and participation in climate-related education and awareness initiatives. Participants also described contributing to sustainable healthcare practices through lower-carbon clinical approaches, environmentally conscious procurement and cross-disciplinary collaboration.

**Conclusions:**

Healthcare workers in private healthcare facilities in Tanzania demonstrate strong awareness of climate change and motivation to support sustainability efforts. However, institutional and structural barriers limit implementation. Efforts to advance sustainable healthcare systems should focus on strengthening organisational support, increasing awareness and enabling healthcare workers to translate knowledge into action.

**STRENGTHS AND LIMITATIONS**

- This study included both clinical and non-clinical healthcare workers from one of Tanzania’s largest private healthcare networks, capturing perspectives across diverse healthcare roles and settings.
- A cross-sectional, self-administered survey enabled data collection from healthcare workers located across multiple facilities within the network.
- The sample size was small relative to the total number of healthcare workers employed within the network, which may limit generalisability.
- Voluntary participation may have introduced self-selection bias, with healthcare workers more interested in climate change and health being more likely to participate.
- The survey was administered only in English, which may have limited participation or comprehension among healthcare workers for whom English is not a first language.

## Background

 The World Health Organization (WHO) has declared climate change the largest global health threat of this century.^1^ Responsible for nearly 5% of global greenhouse gas (GHG) emissions, the healthcare sector is a major contributor to the climate crisis.^2–4^ Examples of sector emissions include those from purchasing goods and services, fossil fuel use in hospitals, medical waste generation and anaesthetic gas use. This creates a feedback loop in which climate-related diseases increase the demand for healthcare services, further raising sector emissions.^2^ Ironically, the institutions tasked with protecting health are themselves contributing to a crisis that endangers it.^5^

Clinical healthcare workers (HCWs), such as physicians and nurses, play a key role in preventive health education. A core competency of these professionals, as outlined by the College of Physicians and Surgeons of Canada, is applying the latest evidence in clinical decision-making.^6^ Research shows that framing climate change as a public health issue makes it more personally relevant and motivates protective and environmentally friendly behaviours.^7^ Patient–practitioner interactions, therefore, present an opportunity for HCWs to raise awareness of climate-related health risks while promoting environmental stewardship within communities. Non-clinical HCWs, such as those in procurement and facility management, also influence sector emissions, as their decisions shape workplace carbon footprints.^4^

Few studies in sub-Saharan Africa (SSA) have examined HCWs’ knowledge of climate change and health, and most focus on specific medical groups, excluding non-clinical staff.^8–11^ Tanzania, among the countries most vulnerable to climate change, remains absent from this research.^8 12^ Given that Global South countries, like Tanzania, face the greatest climate-related health burdens, empowering HCWs to educate, raise awareness and advocate for change in healthcare sector practices provides an avenue to address global inequities.

Family medicine research in SSA highlights that HCWs can lead climate action by modelling climate-friendly practices, invoking intergenerational responsibility and drawing on their exposure to climate-related illness among their patients to engage policy makers and communities.^8^ These findings underscore the untapped potential of HCWs to drive climate action at both individual and systemic levels.

To equip HCWs with the tools to facilitate change, it is first necessary to understand their current knowledge and practices. By identifying gaps, as well as any existing climate action on their part, targeted continuing medical education initiatives can support HCWs in serving as sustainability and climate action advocates. Accordingly, this study aims to assess the perspectives and practices of both clinical and non-clinical HCWs regarding the relationship between climate change and health.^12^ Specifically, the objectives are to evaluate HCWs’ knowledge of climate change and its health impacts, examine their awareness of the healthcare sector’s role in environmental degradation and identify barriers to workplace climate action as well as existing HCW-led initiatives related to environmentally sustainable healthcare.

## Methods

### Study setting

Aga Khan Health Service, Tanzania (AKHST) is an integrated healthcare system that includes the tertiary-level Aga Khan Hospital in Dar es Salaam, the secondary-level Aga Khan Hospital in Mwanza and 26 primary healthcare clinics (called Aga Khan Polyclinics).^13^ With over 90 years of service, AKHST supports national health system strengthening through collaborations with government ministries and academic partnerships with Aga Khan University, Tanzania.

AKHST was selected as the study setting due to its broad geographic coverage, 15 Tanzanian regions with diverse patient populations, and its institutional commitment to achieving net-zero GHG emissions by 2030.^14^ Accordingly, AKHST’s scale and commitment to achieving net-zero emissions created a unique opportunity to assess HCW knowledge and practices in a healthcare system actively working to integrate environmental sustainability into care delivery.

### Study design, population and sample size

A multicentre, cross-sectional design was used to assess HCWs’ climate change and health knowledge via an online, self-administered questionnaire. Eligible participants were all clinical and non-clinical AKHST employees with institutional email accounts. Short-term contract staff without official emails were excluded. Clinical HCWs included physicians, dentists, nurses and allied health professionals; non-clinical HCWs included staff from business development, legal, finance, marketing, food services and facility management departments. A census sampling approach was used, with the survey distributed to all eligible employees, ensuring maximum representativeness. This study is reported using the Strengthening the Reporting of Observational Studies in Epidemiology (STROBE) checklist^15^ (online supplemental file 1).

### Questionnaire development and data collection

The study questionnaire was developed after a review of peer-reviewed and grey literature to align with the study objectives and to draw on published research on climate change and healthcare. Findings from peer-reviewed literature on climate change-related attitudes, perceptions and advocacy among HCWs,^8 9 11^ as well as data published by United Nations agencies, including the WHO, the Intergovernmental Panel on Climate Change and the Office of the United Nations High Commissioner for Refugees, were reviewed to develop questions on the health impacts of climate change.^16–18^ These sources, along with others, informed the development of survey questions examining the broader determinants of health affected by climate change, such as economic strain and insecurity, that influence human health and well-being.

Literature published by organisations and experts in sustainable healthcare and healthcare-related carbon emissions was further used to develop questions assessing HCW awareness of the relationship between healthcare sector activities and carbon emissions.^2–4^ Peer-reviewed literature investigating public perceptions of climate change informed the development of questions aimed at identifying potential climate change misinformation among participants and their primary sources of climate change-related information.^7 10^

To ensure readability and correct interpretation of the questionnaire, the tool was piloted among participants not involved in the study. Feedback was collected, and modifications were made before distributing the tool to the target audience. The final survey (online supplemental file 2) contained 26 questions across four domains: (1) participant demographics, (2) climate change knowledge, (3) climate change and the healthcare sector and (4) barriers and facilitators to climate change adaptation and mitigation in the workplace.

The survey was created in Microsoft Office Forms and distributed electronically to AKHST staff via their institutional Microsoft Office 365 email accounts. Email distribution was selected to ensure accessibility, convenience and broad reach. Participation was voluntary and anonymous. Informed consent was obtained electronically prior to survey completion. The consent form emphasised that participation was entirely voluntary and would have no impact on the respondent’s employment or relationship with AKHST. It outlined confidentiality safeguards, potential risks such as emotional discomfort and possible benefits of participation, including enhanced awareness of climate change. Contact information for the principal investigator was also provided for participants with questions or concerns about the study.

The survey portal opened in November 2023. To maximise participation, reminder emails were sent every few weeks until March 2024, when the survey portal closed. Department heads were encouraged to share the survey link through WhatsApp groups as an additional reminder mechanism. The research team also promoted participation during departmental meetings, where staff could complete the survey and ask questions directly. Duplicate entries were prevented by restricting submissions to one response per email address.

### Data analysis

Data were exported to an Excel sheet with survey respondents being assigned a unique serial number. The collected data were entered, edited and analysed using SPSS V.20. The data also underwent a rigorous cleaning process to check for missing values and inconsistencies before data analysis.

Descriptive statistics, including frequencies and percentages, were calculated to summarise participants’ demographic characteristics, climate change awareness, attitudes, sources of information and related practices. Inferential analysis was conducted using χ^2^ tests to assess associations between categorical variables, such as demographics and awareness levels, as well as perceptions of healthcare facilities’ contributions to climate change. A significance level of p<0.05 was applied for all statistical tests.

### Patient and public involvement

Patients and the public were not involved in the design, conduct, reporting or dissemination of this research study.

## Results

The final online, self-administered cross-sectional survey was distributed to 1100 clinical and non-clinical HCWs through their institutional email addresses. 216 HCWs provided informed consent and completed the questionnaire before the submission deadline, leading to a response rate of 19.64% (216/1100).

### Sociodemographic characteristics

The response rate varied across AKHST facilities, with most respondents (81.9%, n=177) working at Aga Khan Hospital Dar es Salaam. Participants were nearly evenly split by sex, with 102 female (47.2%) and 114 male (52.8%) respondents (table 1). The majority were aged 30–45 years (n=118, 54.6%), and two-thirds were healthcare professionals, primarily physicians, dentists and nurses, while one-third were non-clinical staff. Almost all respondents had completed postsecondary education (n=202, 93.5%), with the majority holding a university degree or higher (n=194, 89.8%). Nearly half had 1–5 years of experience in the healthcare field (n=93, 43.1%).

**Table 1:** Sociodemographic characteristics of study participants (n=216)

Variable	Frequency, n (%)
Sex	
Female	102 (47.2)
Male	114 (52.8)
Age	
<30 years	78 (36.1)
30–45 years	118 (54.6)
46–60 years	16 (7.4)
>60 years	4 (1.9)
Highest level of education	
Secondary	8 (3.7)
Higher secondary	6 (2.8)
Graduate (bachelors)	119 (55.1)
Postgraduate (masters)	70 (32.4)
Doctorate	5 (2.3)
Diploma	8 (3.7)
Occupation	
Doctor (physician or dentist)	56 (25.9)
Pharmacist	8 (3.7)
Nurse	61 (28.2)
Allied health staff	17 (7.9)
Administrative staff	54 (25.0)
Maintenance department staff	2 (0.9)
Other non-clinical healthcare staff	18 (8.3)
Years working in healthcare	
<1 year	19 (8.8)
1–5 years	93 (43.1)
6–10 years	54 (25.0)
11–15 years	30 (13.9)
16–20 years	8 (3.7)
>20 years	12 (5.6)
Type of facility	
Hospital	193 (89.4)
Primary healthcare clinic	23 (10.6)

### HCWs’ views of climate change and health

**Figure 1:**
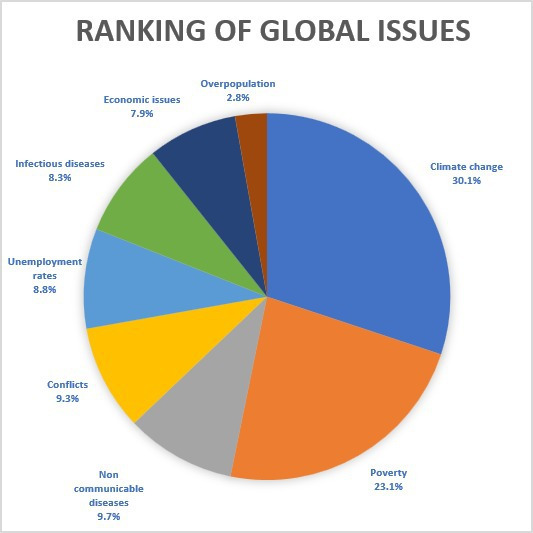
Participant views on the severity of global issues. Participants’ perceived ranking of major global issues, including climate change, poverty, non-communicable diseases, conflicts, unemployment, infectious diseases, economic issues and overpopulation.

Climate change was ranked as the most concerning global issue by 30.1% of respondents, followed by poverty (23.1%), non-communicable diseases (9.7%), conflicts (9.3%), unemployment (8.8%), infectious diseases (8.3%), economic issues (7.9%) and overpopulation (2.8%) (figure 1). Less than 5% of respondents expressed views consistent with climate change misinformation, while 58.8% believed that climate change is a major global concern.

More than 90% of respondents were aware of the relationship between climate change and health, and 73.6% acknowledged health risks as a negative outcome of climate change. Over three-quarters of respondents agreed that climate change poses risks to their health and safety, while 6% disagreed, and 10.6% were unsure. Respiratory diseases were most frequently reported as climate-related health impacts (81.9%), followed by infectious diseases (64.8%) and diarrhoeal diseases (63%). By occupation, 81.1% of physicians and dentists, 87.5% of pharmacists, 80.3% of nurses and 94.1% of allied health staff acknowledged such risks. Among those who reported personal risk, 54.4% (98 of 180) had completed graduate studies as their highest level of education (table 1). The top three reported sources of climate change information were radio/TV/newspapers (67.1%), social media (62%) and the internet (59.7%). However, the most trusted sources were radio/TV/newspapers (60.6%), government agencies (43.5%) and academic journals (38.9%).

**Figure 2:**
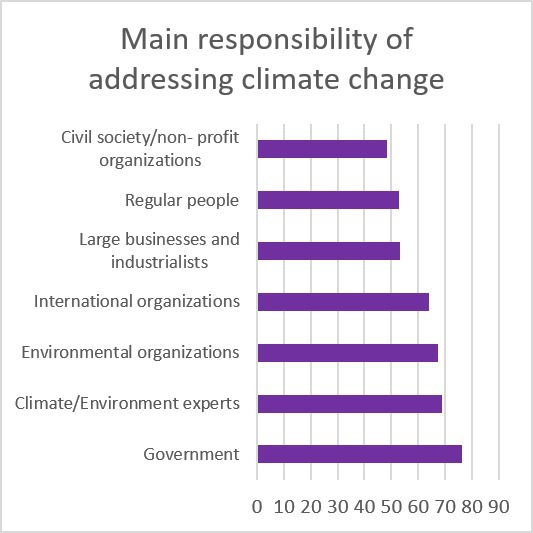
Participant views on climate change responsibility. Participants’ perceptions of the groups or sectors responsible for addressing climate change, including government, climate/environment experts, environmental organisations, international organisations, businesses, individuals and civil society/non-profit organisations.

In terms of responsibility for addressing climate change, 76.4% of respondents identified the government, followed by climate/environmental experts (69%), environmental organisations (67.6%), international organisations (63.9%), large businesses and industrialists (53.2%), regular people (52.8%) and civil society/non-profit organisations (48.6%) as being responsible (figure 2).

### HCWs’ awareness of the environmental impact of healthcare facilities

Almost 80% of respondents agreed that healthcare facilities contribute to climate change. Awareness levels varied, with 26.4% reporting being slightly aware, 34.3% moderately aware, 27.8% aware and 7.4% very aware. No statistically significant associations were observed between awareness and sex, educational attainment, occupation or years of experience in healthcare.

**Figure 3:**
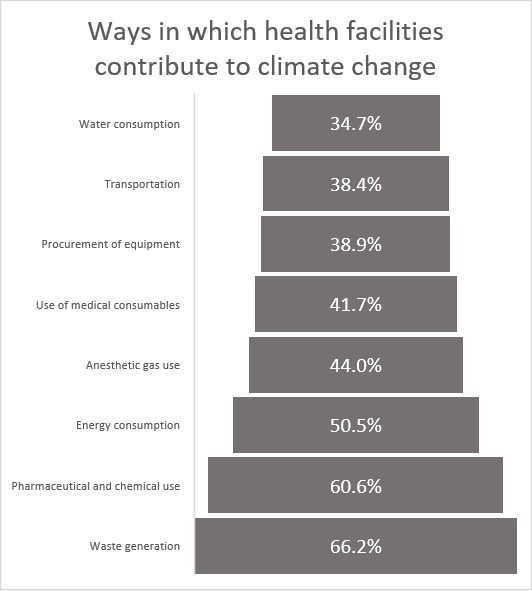
Participant knowledge of the healthcare sector’s contribution to climate change. Participants’ perceptions of the ways in which health facilities contribute to climate change, including waste generation, pharmaceuticals, energy use, anaesthetic gas use, medical consumables, medical and non-medical equipment, transport activities and water consumption.

Waste generation was most frequently identified as a contributor (66.2%), followed by pharmaceutical and chemical use (60.6%) and energy consumption (50.5%). Similarly, no statistically significant relationship was found between occupation or level of education and awareness of the healthcare sector’s environmental impact (figure 3).

### HCW climate action: barriers and exemplars

The main barriers reported by AKHST HCWs when promoting sustainable practices were lack of awareness or concern among colleagues (over 75%), budget constraints (51.4%), resistance to change among colleagues (40.3%) and lack of knowledge (27.3%). Only 13.9% indicated they do not engage in sustainable practices, and 3.7% reported facing no barriers. The activities HCWs were most willing to perform included planting trees (77.3%), recycling (63%) and participating in environmental campaigns (63%). Awareness of AKHST’s Net Zero commitment was reported by 52.3% of respondents. Among those who described their individual contributions to AKHST’s Net Zero commitment (n=86), the most common actions were responsible energy use, proper waste disposal and avoiding single-use plastics. Many provided more than two examples of how they exemplify environmental stewardship in their personal and professional lives (table 2).

**Table 2:** Examples of climate action reported by AKHST HCWs

Theme	Number of responses	Examples of action taken
Waste and water management	53	Segregating and disposing of waste correctly Avoiding single-use plastics Reducing paper use Monitoring and minimising water use Recycling Food waste management
Responsible energy use	32	Switching off lights, AC, and electronic devices when not in use Using renewable sources of energy Promoting sustainable sources of energy
Awareness and education	17	Educating colleagues and AKHST staff Raising patient awareness Educating oneself on the environment and climate change Supporting community awareness initiatives
Contributing to the work of the Net Zero department	15	Collecting facility carbon emissions data Monitoring facility electricity, water and anaesthetic gas use Preparing reports on AKHST Net Zero progress and action items
Personal lifestyle changes	14	Dietary changes Alternative transportation
Sustainable healthcare operations (hospital-level changes)	12	Promoting the use of low-carbon medicines and equipment Reducing the use of high emission anaesthetic gases Collaborating across departments to identify and implement climate-conscious clinical practices
Maintaining their surrounding environment	7	Planting trees Maintaining the cleanliness of green spaces
Workplace policy adherence	6	Abiding by hospital protocols and requirements Following workplace environmental protection directives

Responses were collected from open-text survey answers and categorised into themes. The number of responses per theme indicates how frequently each type of climate action was mentioned. Examples are illustrative and do not include all individual responses.

AC, air conditioning; AKHST, Aga Khan Health Service, Tanzania; HCWs, healthcare workers.

### Differences in climate change awareness between clinical and non-clinical HCWs

Descriptive analysis showed differences in awareness patterns between the two cadres. A higher proportion of non-clinical HCWs demonstrated good awareness of climate change and health (44.6%) compared with clinical HCWs (30.2%), whereas a higher proportion of clinical HCWs demonstrated poor awareness (34.5% vs 23.0%). However, these differences were not statistically significant (p=0.08). Perceptions regarding healthcare facilities’ contribution to climate change were similar between clinical and non-clinical HCWs (p=0.70).

## Discussion

Evaluating HCWs’ baseline climate-health knowledge is a useful first step in developing HCW training programmes and resources. This cross-sectional study aimed to understand the knowledge and practices of clinical and non-clinical HCWs within the AKHST network regarding climate change and health and the role healthcare facilities play in environmental degradation.

Overall, most respondents recognised climate change as a major global issue, with over 90% aware of its impacts on health, and nearly 80% acknowledging the contribution of healthcare facilities to environmental degradation. Key barriers to implementing sustainable practices included a lack of awareness among colleagues and budget constraints, while activities such as responsible energy use and proper waste disposal were examples of common climate action taken by HCWs. These findings provide a foundation for exploring the factors that shape HCWs’ climate-health knowledge and behaviours and for identifying opportunities to strengthen environmentally sustainable practices within healthcare settings.

### HCWs’ views of climate change and health

#### Climate change knowledge

Our findings revealed that HCWs have a good foundational understanding of climate change and acknowledge the threat it poses to human health. Respondents identified multiple health conditions exacerbated by climate change, including communicable and non-communicable diseases and injuries. This is consistent with other similar studies published in the literature investigating knowledge, awareness and/or attitudes regarding climate change and health among HCWs.^8 9 19^ Given that the stigma surrounding mental health in Tanzania is substantial, it was interesting to find that over half of the respondents also selected mental health issues, like depression and anxiety, as a health consequence of climate change. This may be explained by recent climate events, such as the 2024 East African floods that affected tens of thousands of people and caused hundreds of deaths, widespread infrastructure damage and contamination of water supplies.^20 21^ Losing loved ones and valued members of a community can bring about immense feelings of grief, helplessness and despair.^22^ As climate-related disasters increase in their frequency and severity, more communities may be struck with such devastating consequences, thereby seeing or experiencing the mental health implications of climate change firsthand.^23^

#### Climate change misinformation

Very few HCWs expressed scepticism or misinformation about climate change, with only 2.3% believing it to be ‘part of a broader political social agenda’ and 1.9% agreeing that ‘climate change is not a problem’. These results align with studies in South Africa and other Global South settings, where 2%–5% of HCWs disagreed that climate change is a concern.^9 24^ In contrast, studies from the United States of America (USA) reported higher levels of inaccurate beliefs, including the notion that climate change is used as a political tactic and is not an actual threat and that climate change is not actually occurring.^19^ The former studies included HCWs from countries in the global south, regions that bear the greatest burden of climate change. While countries in the global north, including the USA, are responsible for the majority of the world’s emissions, their first-hand experience of climate change is minimal in comparison.^17^ Therefore, this can explain the higher levels of doubt, indicating that much more work needs to be done in equipping the healthcare workforce in the global north with knowledge and skills to support their healthcare sectors in taking accountability.

#### Sources of climate change information

HCWs look to the media for information on climate change. Our study found that media sources were the primary channels through which HCWs accessed climate change information, with 67.1% reporting radio, TV or newspapers as a top source for information and 60.6% indicating their trust in the information provided by these sources. Academic journals were trusted by fewer respondents, reflecting variations in scientific literacy between clinical and non-clinical staff. This is demonstrated by a study done in Minnesota that primarily surveyed healthcare professionals with advanced educational degrees and found that journal articles were the most relied on and recommended source for information on climate change.^19^ Nevertheless, leveraging media channels presents a key avenue for climate advocacy, as previous research has shown thatthe media can promote favourable attitudes towards environmentally-friendly behaviours and that using the media to disseminate accurate information is one way that HCWs can promote climate action.^8 22^

#### Responsibility to address climate change

Regarding responsibility for addressing climate change, 76.4% of respondents identified the government as primarily responsible, followed by climate/environment experts (69%), environmental organisations (67.6%) and international organisations (63.9%). More than half of participants (52.8%) also indicated that regular people share responsibility, highlighting support for collective action against climate change. These findings align with prior research in SSA, where HCWs emphasised coordinated government action as key to addressing climate change and promoting environmental sustainability in healthcare.^8 24^

### HCWs’ awareness of the environmental impact of healthcare facilities

In our study, Tanzanian HCWs demonstrated a clear understanding of the role the healthcare sector plays in environmental degradation. This aligns with findings from studies in France and South Africa, where HCWs acknowledged that more efforts are needed to make healthcare environmentally sustainable.^11 24^ Within our study, the most frequently identified contributors were waste generation and energy use. Similar trends were observed among French HCWs, who also highlighted energy consumption and waste discharges as key environmental impacts.^11^

The prominence of waste and energy as identified contributors may reflect the salience bias, the tendency to focus on more noticeable aspects of one’s environment, and the mere exposure effect, which increases one’s awareness of frequently encountered items or events.^25 26^ For example, filled garbage or medical waste bins and visible electricity use are more easily observed than water consumption, which is often invisible and harder to quantify. Increasing visibility of less obvious contributors to climate change in healthcare settings may help workers understand why systemic changes are necessary and improve acceptance of larger-scale interventions that require HCWs to implement them on the frontline of healthcare facilities. For instance, studies suggest that when physicians are aware of emissions generated by patient travel, they are more likely to follow protocols recommending telemedicine when appropriate, reducing unnecessary in-person visits.^4^

### HCW climate action: barriers and exemplars

#### Barriers towards HCW climate action

According to Kotcher *et al*, only a few studies investigating the barriers that healthcare professionals face in advocating for climate change exist.^9^ Addressing this gap, our study found that HCWs reported several key barriers to translating climate health knowledge into sustainable practices. The most frequently cited obstacles were lack of awareness or concern among colleagues, budget constraints, resistance to change and perceived lack of institutional or leadership support. These findings are consistent with previous studies that found low peer support, lack of hierarchical support and low methodological support as barriers to implementing environmental sustainability within the workplace.^8 9 11^

Interestingly, participants noted a perceived lack of institutional/leadership support despite AKHST’s Net Zero commitment. This mirrors findings from other studies, where healthcare professionals believed that engaging the public on climate and health topics carried professional risk and would have limited colleague support.^9^ These results highlight the importance of fostering a culture of environmental stewardship within healthcare institutions. This is consistent with recommendations from Guihenneuc *et al*, whose study found that establishing sustainable development steering committees can improve transparency of, and staff engagement in, institutional climate commitments.^11^ Similarly, Taylor *et al*, in their commentary, Carbon Foot Printing in Health Systems, emphasise that collective responsibility and decision-making across all levels of a healthcare system are essential for reducing workplace carbon footprints.^27^ Given that many of our participants identified the government and climate/environment experts as primarily responsible for addressing climate change, visible and actionable institutional commitment is critical to empower HCWs to engage in sustainable practices.

#### Exemplars of HCW climate action

Of the 113 respondents aware of AKHST’s Net Zero commitment, 86 of them provided examples of how they contributed to this initiative. Notably, 31 respondents provided more than two examples of climate action in their workplace and personal lives. These responses were categorised into 13 themes (table 2).

##### Waste management and energy use

Proper waste and water management was the most frequently reported theme (n=53), with common practices including waste segregation, reducing waste production and recycling. This is positive to note, given that reducing medical and material waste has been recognised as a key green intervention to lower GHG emissions.^4^ Responsible energy use was the second most cited theme (n=32). Examples included reducing electricity consumption by turning off lights, air conditioning and devices when not in use, which are also ways that French HCWs reduce electricity consumption at the workplace.^4^ Examples of nature-based interventions for climate action were also offered by participants under this theme, including using sunlight instead of electricity and promoting renewable energy sources, such as solar-powered electricity, to reduce carbon emissions. This finding is also promising, given that fossil-fuelled electricity and gas are among the main sources of CO₂ emissions in healthcare services and interventions, such as installing solar panels and monitoring energy use, can effectively reduce emissions.^4^

##### Awareness and education

Responses under the theme of awareness and education (n=17) included educating patients, reminding colleagues of sustainability responsibilities and raising awareness about climate change and its health consequences. The literature shows that even though healthcare professionals may understand the link between climate change and health, many report feeling their knowledge is inadequate, which can limit their engagement in climate action, thus pointing to the need for HCWs to take part in ongoing training on these topics.^9^ Continuing professional education is helpful, with 76% of participants in Kotcher *et al*.’s multinational study demonstrating support for further training on climate change and health.^9^ In this viewpoint, the active efforts by HCWs in the study indicate a solid foundation on which targeted training initiatives can be built.

##### Hospital-level changes

Several studies have shown that a patient’s treatment has a significant environmental impact. This impact depends on factors such as in-person versus telemedicine care, the use of invasive technology, prescribed medication, ongoing treatment coordination, efficient versus inefficient referral pathways and protocols for preventing overtreatment.^4^ Similar initiatives in other healthcare systems have demonstrated that structured collaboration across roles can result in measurable reductions in emissions while maintaining care quality.^4^ These findings present an opportunity for clinical and non-clinical staff to work together to optimise care pathways and management practices, improving sustainability without compromising patient safety or quality of care.

Survey responses indicate that AKHST HCWs are already implementing many of these practices independently, highlighting their potential as advocates for environmentally sustainable healthcare. For example, suggestions to source items from suppliers with lower emissions align with evidence that mandatory supplier environmental requirements could substantially reduce emissions from pharmaceutical and non-pharmaceutical procurement.^4^ Participants also reported efforts to reduce nitrous oxide, switch to lower-emission alternatives and use low-carbon medicines, such as the inhaler sevoflurane. Importantly, respondents emphasised that multidisciplinary collaboration, such as meetings between procurement staff, pharmacists and anaesthesiologists, is essential to successful sustainability interventions, and many expressed a willingness to take part in such efforts. Accordingly, the unique contribution of this study lies in the inclusion of non-clinical HCWs, whose perspectives reveal additional pathways for hospitals to reduce their environmental impact. Future research should further explore how to coordinate non-clinical and patient-facing roles to enhance care pathways and advance more sustainable operational models.

### Perspectives of clinical versus non-clinical HCWs

A unique aspect of this study was the inclusion of both clinical and non-clinical HCWs. The higher proportion of good awareness levels observed among non-clinical HCWs may be related to the nature of their roles within healthcare facilities. Non-clinical HCWs often work across administrative, operational, environmental or logistical functions, which may provide broader exposure to institutional sustainability practices, environmental policies and climate-related operational challenges. In contrast, clinical HCWs may be more primarily focused on direct patient care responsibilities, potentially limiting engagement with broader environmental and system-level issues. However, further research is needed to better understand the factors underlying these differences between HCW cadres.

### Strengths and limitations

This study adds to the existing body of literature on the knowledge of HCWs regarding climate change, its impact on health and the contribution of the healthcare sector in this global crisis. By surveying clinical and non-clinical healthcare staff working at one of Tanzania’s largest private healthcare networks, we captured a range of perspectives from HCWs practising across different socioeconomic and cultural contexts throughout the country.

Although this study was conducted among HCWs in Tanzania, and findings may be influenced by the country’s specific sociocultural, economic and environmental context, several results may be relevant to other low- and middle-income countries, particularly in SSA, where healthcare systems face similar climate-related vulnerabilities and resource constraints. Previous studies from other regions have reported similar findings regarding HCWs’ recognition of climate change as a major health concern, support for coordinated government and institutional action and awareness of the healthcare sector’s environmental impact. However, differences in healthcare infrastructure, climate exposure, educational systems and sociopolitical contexts may limit direct generalisability to high-income settings. As such, findings should be interpreted within the Tanzanian context while also contributing to the growing global evidence base on HCWs’ perspectives regarding climate change and health.

This present study also has some limitations that must be acknowledged. First, this study had a small sample size (n=216) compared with the actual number of clinical and non-clinical HCWs within the AKHST network. It is possible that those interested in climate change and health were motivated to participate. Therefore, our findings may not reflect those of the entire network. Next, many of the questions allowed participants to select more than one response. It is possible that some respondents selected multiple answers because they were unsure about the correct choice to make. Since the survey was only offered in English and no version was available in Kiswahili, Tanzania’s official language, it is also possible that some participants faced language barriers when completing the survey.

## Conclusion

This study underscores the evolving role of HCWs, not only as care providers, but also as critical agents of change in the fight against the global climate crisis. By exploring their perceptions and practices within a private healthcare system, we reveal both the awareness and the gaps that exist in integrating environmental sustainability into clinical and institutional routines. The findings demonstrate that while many HCWs recognise the health implications of climate change, actionable engagement remains limited by structural, educational and policy-related barriers.

Nevertheless, many HCWs were willing to adopt climate-friendly behaviours, highlighting the potential for this group to become advocates for environmental sustainability within healthcare and beyond. To tap into this potential, ongoing professional development, enhanced institutional accountability and collaboration across professions are essential. By integrating climate change and sustainability into the professional culture of healthcare institutions in Tanzania and similar settings, the sector could not only reduce its environmental footprint, but also strengthen its core mission of protecting human health in a changing climate.

The insights gathered here provide a foundation for designing targeted interventions and policies that align healthcare delivery with climate resilience. The healthcare sector ultimately has both the moral imperative and the strategic capacity to lead by example in mitigating climate change and protecting public health.

## Supplementary material

10.1136/bmjopen-2026-117293 online supplemental file 1

10.1136/bmjopen-2026-117293 online supplemental file 2

## Data Availability

Data are available upon reasonable request.
