# Supplementary material for: Healthcare workers’ views and actions on climate change and health in private healthcare facilities in Tanzania: a cross-sectional study
Source: BMJ Open. 2026 Jul 28;16(7):e117293. doi: 10.1136/bmjopen-2026-117293 (PMC13423065; doi:10.1136/bmjopen-2026-117293)
Supplement: online supplemental file 1 [file bmjopen-16-7-s001.pdf]

Appendix 1: STROBE Statement—Checklist of Items for Cross-Sectional Studies

**Title and Abstract**			
**Item**	**Recommendation**	**Reported on Page No.**	**Description**
1a. Title	Indicate the study's design with a commonly used term in the title or the abstract	Page 1	Cross-sectional study stated
1b. Abstract	Provide in the abstract an informative and balanced summary of what was done and what was found	Page 1	Balanced summary provided

**Introduction**			
**Item**	**Recommendation**	**Reported on Page No.**	**Description**
2. Background/ rationale	Explain the scientific background and rationale for the investigation being reported	Page 3	Climate-health context described
3. Objectives	State specific objectives, including any prespecified hypotheses	Page 3	Aim clearly stated

**Methods**			
**Item**	**Recommendation**	**Reported on Page No.**	**Description**
4. Study design	Present key elements of study design early in the paper	Page 4	Cross-sectional design stated
5. Setting	Describe the setting, locations, and relevant dates, including periods of recruitment, exposure, follow-up, and data collection	Page 3	Private facilities in Tanzania
6a. Participants	Give the eligibility criteria, and the sources and methods of selection of participants	Page 4	Clinical and non-clinical staff included
7. Variables	Clearly define all outcomes, exposures, predictors, potential confounders, and effect modifiers. Give diagnostic criteria, if applicable	Page 4	Knowledge, attitudes, and practices
8. Data sources/ measurement	For each variable of interest, give sources of data and details of	Page 4	Structured questionnaire

	methods of assessment (measurement). Describe comparability of assessment methods if there is more than one group		
9. Bias	Describe any efforts to address potential sources of bias	Page 12	Potential biases acknowledged under study limitations
10. Study size	Explain how the study size was arrived at	Page 5	Sample size described
11. Quantitative variables	Explain how quantitative variables were handled in the analyses. If applicable, describe which groupings were chosen and why	Page 5	Variables categorized
12a. Statistical methods	Describe all statistical methods, including those used to control for confounding	Page 5	Frequencies and percentages
12b. Subgroups	Describe any methods used to examine subgroups and interactions	Page 5	Clinical vs non-clinical comparisons
12c. Missing data	Explain how missing data were addressed	N/A	N/A
12d. Sampling strategy	If applicable, describe analytical methods taking account of sampling strategy	Page 4	Convenience sampling
12e. Sensitivity analyses	Describe any sensitivity analyses	N/A	N/A

**Results**			
**Item**	**Recommendation**	**Page**	**Description**
13a. Participant numbers	Report numbers of individuals at each stage of study—eg numbers potentially eligible, examined for eligibility, confirmed eligible, included in the study, completing follow-up, and analysed	Page 5	Response rate reported

13b. Non-participation	Give reasons for non-participation at each stage	Page 5, 12	Non-response acknowledged
13c. Flow diagram	Consider the use of a flow diagram	N/A	Not included
14a. Descriptive data	Give characteristics of study participants (eg demographic, clinical, social) and information on exposures and potential confounders	Pages 5-6	Table 1: Sociodemographic characteristics of study participants
14b. Missing data	Indicate number of participants with missing data for each variable of interest	N/A	N/A
15. Outcome data	Report numbers of outcome events or summary measures	Pages 6-9	Knowledge, attitudes, and practices findings
16a. Main results	Give unadjusted estimates and, if applicable, confounder-adjusted estimates and their precision (eg, 95% confidence interval). Make clear which confounders were adjusted for and why they were included	Pages 6-9	Frequencies and percentages
16b. Category boundaries	Report category boundaries when continuous variables were categorized	Page 6-9	Definitions provided
16c. Translation of estimates	If relevant, consider translating estimates of relative risk into absolute risk for a meaningful time period	N/A	N/A

17. Other analyses	Report other analyses done—eg analyses of subgroups and interactions, and sensitivity analyses	Pages 7,9	Subgroup analyses

**Discussion**			
**Item**	**Recommendation**	**Page**	**Description**
18. Key results	Summarise key results with reference to study objectives	Page 9-12	Summary of findings
19. Limitations	Discuss limitations of the study, taking into account sources of potential bias or imprecision. Discuss both direction and magnitude of any potential bias	Page 12	Bias and constraints discussed
20. Interpretation	Give a cautious overall interpretation of results considering objectives, limitations, multiplicity of analyses, results from similar studies, and other relevant evidence	Pages 12	Findings contextualized
21. Generalisability	Discuss the generalisability (external validity) of the study results	Page 12	External validity discussed

**Other Information**			
**Item**	**Recommendation**	**Page**	**Description**
22. Funding	Give the source of funding and the role of the funders for the present study and, if applicable, for the original study on which the present article is based	Page 13	Funding statement included
