# Supplementary material for: Healthcare workers’ views and actions on climate change and health in private healthcare facilities in Tanzania: a cross-sectional study
Source: BMJ Open. 2026 Jul 28;16(7):e117293. doi: 10.1136/bmjopen-2026-117293 (PMC13423065; doi:10.1136/bmjopen-2026-117293)
Supplement: online supplemental file 2 [file bmjopen-16-7-s002.pdf]

Climate Change Perceptions and Practices of AKHST Healthcare Workers

Nov 7, 2023

The purpose of this survey is to understand the level of climate change knowledge that AKHST staff hold, as well as any environmentally friendly actions they take. The information collected can be used to determine ways that the healthcare sector can contribute to climate change adaptation and mitigation strategies.

* Required

* This form will record your name, please fill your name.

Letter of Information for Informed Consent

VOLUNTARY PARTICIPATION

Your participation in this intervention study is **entirely voluntary.** This means that whether or not you choose to participate in this study, there will be no effect on your relationship with AKHD or AKHST. There is no financial compensation for your participation in this research.

CONFIDENTIALITY

All research records obtained in this study will be kept strictly confidential. All of the data collected via survey and interviews will only be accessible to the principal investigator and the research team. The results of the study may be published for scientific purposes, but it will not include any identifiable references to you.

POSSIBLE RISKS

While unlikely, there is the potential that you may experience uncomfortable emotions while completing the survey, such as feeling guilty for not knowing about climate change.

POSSIBLE BENEFITS

The opportunity to become more aware about climate change and gain motivation to take positive climate action, as well as the positive impact on health and well-being that the long-term favourable environmental outcomes associated with this research can bring, are some benefits of your participation within this study.

1. What is your age? *

- Young (<30 years)
- Early Adulthood (30-45 years)
- Middle Age (46-60 years)
- Old Age (>60 years)

2. What is your gender? *

- Man
- Woman

3. What is your highest level of education? *

- Secondary
- Higher Secondary
- Graduate
- Postgraduate
- Doctorate
- Other

4. What is your occupation? *

- Doctor (e.g., Physician or Dentist)
- Pharmacist
- Nurse
- Allied health care professional (e.g., physiotherapist, technician, technologist etc.)
- Administrative staff (e.g., human resources, procurement, finance, legal etc.)
- Maintenance staff (e.g., Engineering, housekeeping etc.)
- Other non-clinical
- Other

5. How many years have you been working in healthcare? *

- Less than 1 year
- 1-5 years
- 6-10 years
- 11-15
- 16-20
- More than 20 years

6. What type of healthcare facility do you work in? *

- Hospital
- Outreach Centre

7. Which Aga Khan health facility do you specifically work in? *

- ◯ Aga Khan Hospital Dar es salaam
- ◯ Aga Khan Hospital Mwanza
- ◯ Aga Khan Health Polyclinic Arusha
- ◯ Aga Khan Health Polyclinic Bukoba
- ◯ Aga Khan Health Polyclinic Bunda
- ◯ Aga Khan Health Polyclinic bunju
- ◯ Aga Khan Health Polyclinic Dialysis Centre
- ◯ Aga Khan Health Polyclinic Dodoma
- ◯ Aga Khan Health Polyclinic Geita
- ◯ Aga Khan Health Polyclinic Iringa
- ◯ Aga Khan Health Polyclinic Kahama
- ◯ Aga Khan Health Polyclinic Kibaha
- ◯ Aga Khan Health Polyclinic Kigamboni
- ◯ Aga Khan Health Polyclinic Kimara
- ◯ Aga Khan Health Polyclinic masaki
- ◯ Aga Khan Health Polyclinic Mbagala
- ◯ Aga Khan Health Polyclinic Mbeya
- ◯ Aga Khan Health Polyclinic Mbezi
- ◯ Aga Khan Health Polyclinic Morogoro
- ◯ Aga Khan Health Polyclinic Tabata
- ◯ Aga Khan Health Polyclinic Tabora
- ◯ Aga Khan Health Polyclinic Tandika
- ◯ Aga Khan Health Polyclinic Tanga
- ◯ Aga Khan Health Polyclinic Town Centre
- ◯ Aga Khan Health Polyclinic Sinza
- ◯ Aga Khan Health Polyclinic Ukonga
- ◯ Aga Khan Health Polyclinic Yombo
- ◯ Aga Khan Health Polyclinic Zanzibar

8. Based on your opinion, please rank the following global issues in order of most to least concerning (i.e., the most important issues will be higher and less important issues will be lower). *

- Non-communicable disease
- Overpopulation
- Poverty
- Conflicts (e.g., terrorism, violence, war, crime)
- Infectious diseases
- Global economic issues
- Climate change
- National unemployment rates

9. Have you heard about climate change and its impact on health? *

- Yes
- No
- I'm not sure

10. How would you rate your awareness of the impact that healthcare facilities have on the environment and climate? *

	Not aware	Slightly aware	Moderately aware	Aware	Very aware
Awareness level	◯	◯	◯	◯	◯

11. Which of the following best expresses your belief about the topic of climate change? *

- ◯ Climate change is not a problem.
- ◯ Climate change is a natural phenomenon.
- ◯ Climate change is a major issue of global concern.
- ◯ Climate change is a topic that is part of a broader political social agenda.
- ◯ Climate change is the result of human activities.
- ◯ Climate change doesn't concern me.

12. Do you think climate change threatens your personal health and safety? *

- ◯ Yes
- ◯ No
- ◯ I'm not sure

13. If the answer above is yes, please briefly describe how climate change threatens your personal health and safety. If you answered no, please skip to question 14.

14. From where have you learned or heard about climate change? Select all that apply. *

- [ ] Personal experience(s)
- [ ] Family, friends and/or colleagues at work
- [ ] Radio, television and/or newspapers
- [ ] The internet
- [ ] Social media platforms
- [ ] School, college or university
- [ ] Government agencies
- [ ] Academic journals
- [ ] Healthcare provider(s)
- [ ] Other

15. which sources of climate change information do you trust? *

- [ ] Personal experience(s)
- [ ] Family, friends and/or colleagues at work
- [ ] Radio, television and/or newspapers
- [ ] The internet
- [ ] Social media platforms
- [ ] School, college or university
- [ ] Government agencies
- [ ] Academic journals
- [ ] Healthcare provider(s)
- [ ] Other

16. Which of the following cause climate change? *

- [ ] Increased use of fossil fuels
- [ ] Deforestation
- [ ] Rapid industrialization
- [ ] Transportation
- [ ] Pollution
- [ ] Urbanization
- [ ] None of the above

17. Which of the following are a result of climate change? *

- [ ] Droughts
- [ ] Floods
- [ ] Temperature extremes
- [ ] Rising sea levels
- [ ] Unpredictable weather patterns
- [ ] An increase in extreme weather events
- [ ] Land degradation
- [ ] Loss of wildlife and vegetation
- [ ] Health risks
- [ ] Food insecurity
- [ ] Conflict over resources

18. Do you think healthcare facilities contribute to climate change? *

- ○ Yes
- ○ No
- ○ I'm not sure

19. If you answered yes to question 18, please select the ways in which you believe healthcare facilitates contribute to climate change. If you answered no, skip to question 20.

- [ ] Energy use
- [ ] Water consumption
- [ ] Activities requiring transportation
- [ ] Waste generation
- [ ] Anaesthetic gas use
- [ ] Pharmaceuticals and chemicals
- [ ] Medical consumables
- [ ] The use of medical and non-medical equipment

20. What are health impacts of climate change? *

- [ ] Respiratory disease (e.g., asthma)
- [ ] Infectious disease (e.g., malaria)
- [ ] Malnutrition (e.g., undernourishment, obesity etc.)
- [ ] Diarrheal disease (e.g., cholera)
- [ ] Mental health issues (e.g., anxiety, depression etc.)
- [ ] Non-communicable disease (e.g., cancer)
- [ ] Injuries (e.g., drownings, accidents etc.)

21. Of the following options, who do you believe has the main responsibility of addressing climate change? *

- [ ] Regular people
- [ ] The government
- [ ] Large businesses and industrialists
- [ ] Civil society/non-profit organizations
- [ ] Climate/environment experts
- [ ] Environmental organizations
- [ ] International organizations (e.g., The United Nations, The World Health Organization)
- [ ] Other

22. What are the main barriers or challenges that you have encountered when trying to promote environmentally sustainable practices within your healthcare facility? *

- [ ] Lack of awareness and/or concern among colleagues
- [ ] Budget constraints
- [ ] Resistance to change among colleagues
- [ ] Lack of institutional/leadership support
- [ ] My lack of knowledge on the subject
- [ ] N/A. I don’t promote environmentally sustainable practices at work
- [ ] I face no barriers or challenges
- [ ] Other

23. Of the following activities, which would you perform to take action against climate change? *

- [ ] Walking or cycling to and from work
- [ ] Using public transportation to get to and from work
- [ ] Monitoring water use
- [ ] Reducing electricity use
- [ ] Reducing food waste
- [ ] Recycling
- [ ] Planting trees
- [ ] Participating in environmental campaigns
- [ ] Using renewable sources of energy
- [ ] Avoiding single-use plastics (e.g., plastic water bottles, plastic bags)
- [ ] Other

24. Are you aware of the AKHST Net Zero and Environmental Sustainability Commitment? *

- ○ Yes
- ○ No

25. If you answered yes to question 24, please explain how you are contributing to this initiative?
If you answered no, please skip to question 26.

26. The information you have provided will be used to understand the climate change knowledge and practices across the AKHST.

    Your personal information will not be visible anywhere and strict confidentiality will be maintained by the research team. The institutional benefits of this study is the potential to identify areas that can be improved so as to better support AKHST in reaching AKDN's Net Zero by 2030 commitment. Therefore, you are responsible for responding to all questions honestly because wrong information will not help the institution.

    If you would like a longer version of the study's letter of information consent form, or if you have any questions, please contact the Principal Investigator, Prof. Ahmed Jusabani, by phone at 0713273890 or by email at ahmed.jusabani@akhst.org.

    *

    By selecting yes to the question below, you agree that you have read and understood the consent information provided and that you willingly volunteer to participate in this research study.

    ◯ Yes

This content is neither created nor endorsed by Microsoft. The data you submit will be sent to the form owner.

Microsoft Forms
