# Supplementary material for: Healthcare workers’ views and actions on climate change and health in private healthcare facilities in Tanzania: a cross-sectional study
Source: BMJ Open. 2026 Jul 28;16(7):e117293. doi: 10.1136/bmjopen-2026-117293 (PMC13423065; doi:10.1136/bmjopen-2026-117293)
Supplement: Supplementary file 3 [file bmjopen-2026-117293.reviewer_comments.pdf]

PEER REVIEW HISTORY

BMJ Open publishes all reviews undertaken for accepted manuscripts. Reviewers are asked to complete a checklist review form and are provided with free text boxes to elaborate on their assessment. These free text comments are reproduced below.

ARTICLE DETAILS

Title (Provisional)

Healthcare Workers' Views and Actions on Climate Change and Health in Private Healthcare Facilities in Tanzania: A Cross-Sectional Study

Authors

Kara, Sophia Lubna; Jusabani, Ahmed M.; Premji, Elishah; Abeid, Muzdalifat

---

VERSION 1 - REVIEW

**Reviewer**	**1**
**Name**	**Thompson, Hannah**
**Affiliation**	**Icahn School of Medicine at Mount Sinai**
**Date**	**12-Mar-2026**
**COI**	**None**

I commend the authors on their work. They performed a cross-section study evaluated perceptions of climate change amongst healthcare workers in Tanzania. As we learn about the impacts of climate change on health, it is critical to involve healthcare workers who are on the frontlines. There currently is limited knowledge on this topic, and this study adds to the literature. I had a few comments:

Would the authors expound upon survey development? It may be helpful to cite the literature used to create the survey.

In the results, it would be helpful to have the survey response rate.

In the “Strengths and Limitations” section, would the authors be able to comment on the generalizability of their data? I am particularly wondering around how these results may compare to other countries in Africa or in the world.

Reviewer **2**

Name **Oduwole, Elizabeth**

Affiliation **University of Stellenbosch Department of Interdisciplinary Health Sciences, Global Health**

**Date**	**24-Apr-2026**
**COI**	**None**

I applaud your effort in conducting this interesting and timely research and would like you to consider the following points when reviewing the article before the final submission.

1. Line 34, page 5, states that reminders were sent every 15 days till March 2024, when the survey portal closed. When was the survey portal opened, and for how long? Please specify the exact length of the data collection period.

2. Under data analysis, the use of means and standard deviation, where applicable, was mentioned, but frequencies and percentages were the only descriptive statistics reported in the manuscript. If means and SD were not used, kindly remove their mention in the manuscript.

3. Line 49, page 4, mentions tertiary, secondary, and primary healthcare clinics, but the last line in Table 2 states polyclinic. Kindly clarify and be consistent in the use of the nomenclature for the health facilities.

4. One of the unique aspects of the study emphasized by the authors is the inclusion of the perspectives of non-clinical health workers. But this was not visible in the results and discussion section. It would be good to highlight perspectives unique to non-clinical health workers that are lacking among clinical health workers, and vice versa.

5. Include the questionnaire as a supplementary document to the manuscript for publication to enhance study reproducibility.

6. Do cite the STROBES checklist (in the methods section) and include it in the reference list as requested by the developers.

I wish you all the best in this and other endeavours

VERSION 1 - AUTHOR RESPONSE

Reviewer 1: Dr. Hannah Thompson, Icahn School of Medicine at Mount Sinai

Comment 1: Would the authors expound upon survey development? It may be helpful to cite the literature used to create the survey.

- **Response:** We expanded the Methods section to provide a more detailed description of the survey development process. The questionnaire was informed by literature on healthcare workers' knowledge, attitudes, and practices regarding climate change and health, adapted to the Tanzanian context, and pre-tested for clarity and relevance.

Comment 2: In the results, it would be helpful to have the survey response rate.

- **Response:** The response rate has now been included in the Results section, including the

number invited, number responding, and the calculated response percentage.

Comment 3: Would the authors comment on the generalizability of their data?

- **Response:** We expanded the Strengths and Limitations section to discuss generalizability. We acknowledge that findings from private healthcare facilities in Tanzania may not directly apply to all settings, although they align with emerging evidence from other low- and middle-income countries.

Reviewer 2: Mrs. Elizabeth Oduwole, University of Stellenbosch Department of Interdisciplinary Health Sciences, Cochrane South Africa

Comment 1: Specify the exact length of the data collection period.

- **Response:** The Methods section now specifies the survey opening date, closing date, total duration of data collection, and reminder schedule.

Comment 2: Remove mention of means and standard deviations if not used.

- **Response:** The Data Analysis section has been revised to remove mention of means and standard deviations since only frequencies and percentages were used.

Comment 3: Clarify nomenclature of health facilities.

- **Response:** Terminology has been standardized throughout the manuscript using primary, secondary, and tertiary healthcare facilities categories.

Comment 4: Highlight perspectives unique to non-clinical health workers.

- **Response:** The Results and Discussion sections now explicitly compare perspectives between clinical and non-clinical healthcare workers.

Comment 5: Include the questionnaire as a supplementary document.

- **Response:** The questionnaire has now been included as a supplementary file to improve transparency and reproducibility.

Comment 6: Cite the STROBE checklist and include it in the reference list.

- **Response:** The STROBE guidelines have now been cited in the Methods section, added to the references, and the checklist included as supplementary material.

We would also like to note that the manuscript currently exceeds *BMJ Open*'s word count limit. This is because the manuscript was originally submitted to *BMJ Global Health*, which permits a higher word count. Following editorial assessment, the manuscript was transferred to *BMJ Open*, and we were encouraged to expand certain sections to improve clarity and completeness. As a result, the revised manuscript length increased further. We hope the editors will consider this context when assessing the submission, as we believe the additional detail enhances the transparency and overall quality of the work.

We confirm that this manuscript is original, has not been published previously, and is not under consideration elsewhere. All authors have approved the final version and agree to its submission to *BMJ Open*. Thank you for your time and consideration. We look forward to your response.
